# Individual, Prenatal, Perinatal, and Family Factors for Anxiety Symptoms Among Preschool Children

**DOI:** 10.3389/fpsyt.2021.778291

**Published:** 2021-12-20

**Authors:** Xiuxiu Ding, Jun Wang, Ning Li, Wanying Su, Hao Wang, Qiuxia Song, Xianwei Guo, Mingming Liang, Qirong Qin, Liang Sun, Mingchun Chen, Yehuan Sun

**Affiliations:** ^1^Department of Epidemiology and Health Statistics, School of Public Health, Anhui Medical University, Hefei, China; ^2^Department of Maternal, Child and Adolescent Health, School of Public Health, Anhui Medical University, Hefei, China; ^3^Ma'anshan Center for Disease Control and Prevention, Ma'anshan, China; ^4^Fuyang Center for Disease Control and Prevention, Fuyang, China; ^5^Changfeng Center for Disease Control and Prevention, Changfeng, China

**Keywords:** anxiety, preschool, child, left-behind, prenatal

## Abstract

Anxiety is one of the most common psychological disorders among children. Few studies have investigated the prevalence and comprehensive factors for anxiety among preschool children in China. This study aimed to assess the prevalence of anxiety and explore influential factors at multiple levels including individual, prenatal and perinatal, and family factors, associated with anxiety symptoms among preschool children. The multisite cross-sectional study was conducted in Anhui Province and included 3,636 preschool children aged 3–6 years. Anxiety symptoms of children were assessed using the Chinese version of the Spence Preschool Anxiety Scale. Logistic regression analyses were performed to explore associations between factors at multiple levels and significant anxiety symptoms, and the model was validated internally using 10-fold cross-validation. Among the participants, 9.1% of children had significant anxiety symptoms. Girls reported more significant anxiety symptoms. Children's poor dietary habits, sleep disturbances, autistic tendencies, and left-behind experience; maternal poor prenatal emotional symptoms; and more caregivers' anxiety symptoms were significantly associated with anxiety symptoms among children. The result of 10-fold cross-validation indicated that the mean area under the curve, sensitivity, specificity, and accuracy were 0.78, 70.45%, 78.18%, and 71.15%, respectively. These factors were slightly different among different subtypes of anxiety symptoms. The results of this study suggested that anxiety symptoms in preschool children were prevalent, particularly in girls. Understanding early-life risk factors for anxiety is crucial, and efficient prevention and intervention strategies should be implemented in early childhood even pregnancy.

## Introduction

Anxiety is one of the most common psychological disorders among children and is associated with serious academic and social impairments and even adulthood psychological problems (1–4). Studies have demonstrated that the anxiety age of onset is approximately 6 years old (4–6). Preschool children's anxiety is generally moderately stable and tends to persist into childhood and adolescence (7, 8). A longitudinal study from Canada has indicated that 14.7% of preschool children maintain constantly elevated anxiety symptoms from 1.5 to 5.0 years of age (9). One recent study exploring the trajectories of anxiety symptoms from preschool to school age has suggested that high stable anxiety beginning at preschool age is associated with lower school engagement, poorer peer relations, and greater functional impairment during the later school age (10). Researchers have proposed that preschool age may be an optimal developmental window for anxiety prevention or early intervention (11).

Currently, considerable research efforts have been made to improve knowledge surrounding the aetiology of anxiety, which is believed to be affected by genetic vulnerability and environmental factors (10, 12–18). Previous classical twin studies point genetic background as the predominant factor for the emergence of anxiety-related behaviours (19), while a 30–40% heritability is reported (20). Individual internal characteristics such as age, gender, and children's emotional and behavioural characteristics are involved in susceptibility to anxiety. Some studies have shown that younger children report higher anxiety symptoms than older children (21, 22). Regarding gender differences, the results show a relatively incongruent picture in previous research (2, 22–24). Children's behavioural problems such as sleep disturbances, depression, high behavioural inhibition, attention-deficit hyperactivity disorder symptoms, and peer victimisation seem to predict follow-up anxiety (3, 10, 25). The influence of external environmental factors throughout foetal and early childhood involving prenatal, perinatal, and parental and family factors cannot be underestimated (12, 26, 27). With the development of the developmental origins of adult disease (DOHaD) theory (28), researchers traced these factors back to the pregnancy period according to the foetal programming mechanisms (29, 30). Evidence from cohort studies has indicated that preterm birth, caesarean section, hypertensive disorders of pregnancy, prenatal and postnatal anxiety, depression, and distress increase the risk of offspring anxiety (14, 27, 31–33). Given that the family exerts important influence on a child's development, researchers have noted the role of family factors in child anxiety. Some adverse family factors such as parental corporal punishment and abuse are positively associated with childhood anxiety (16, 34). Additionally, children who were separated from parents too early have more anxiety symptoms (17). In addition, one study has proposed a novel evidence for environmental transmission, and the anxiety of parents and children may influence each other in different ways (12). Severe early adversities may trigger chronic and neurobiologically distinct internalising symptoms starting from the preschool period (15). Therefore, it is of great importance to understand the impacts of early-life risk factors on children's anxiety. Thus far, numerous studies surrounding these issues are predominantly focused on school-aged children and from Western countries. However, only a few studies with small sample sizes and exploring only limited factors have been conducted among preschool children in China (35–37).

Taken together, the findings from previous research show that it is critical to better understand anxiety symptoms during the preschool period and its potential risk factors to minimise the negative effects on these children. In the current study, we hypothesised that exposure to prenatal and perinatal adversities, individual problematic behaviours, and adverse family environmental factors from pregnancy to early childhood might increase the risks of preschool children's anxiety. This study aims to ascertain whether comprehensive factors including individual, prenatal, perinatal, and family factors are associated with anxiety among preschool children and provide scientific evidence supporting the use of early-life interventions based on multisite cross-sectional survey.

## Materials and Methods

### Participants

This multisite cross-sectional study was conducted in 26 kindergartens of four counties including Funan county in Fuyang City, Changfeng county and Fexi county in Hefei City, and Bowang district (same as county administrative level) in Ma'anshan City, located in the north, middle, and south of Anhui Province from September 2019 to January 2020. A total of 3,802 questionnaires were distributed in the initial investigation. Finally, 3,636 effective questionnaires were collected with a total response rate of 95.6%, after checking for completeness and logic. Participants, including 3,636 preschool children aged 3–6 years and their primary caregivers, voluntarily participated in the investigation. Children and their caregivers with cognitive impairment, deafness, or language communication barriers and children with severe mental or physical illnesses were excluded. Prior to data collection, caregivers' consent was obtained. Then, caregivers were invited to complete a structured questionnaire through a face-to-face interview. The study was approved by the Ethics Committee of Anhui Medical University (20180402), and confidentiality was assured.

### Independent Variables

#### Individual Characteristics

Individual characteristics included children's age, gender, body mass index (BMI), dietary habits, sleep disturbances, and autistic tendencies. Children's age and gender were collected by using a questionnaire based on primary caregivers' self-reports. Children's BMI was calculated by dividing the weight (kg) by height (m) squared (weight and height measured by investigators), and the World Health Organization Child Growth Standards for age- and sex-specific cutoffs were employed (38). Dietary habits of preschool children were assessed by caregivers' reports using the adapted Identification and Management of Feeding Difficulties scale (IMFeD) (39), which consists of 17 items, and higher total scores indicate better dietary habits. Cronbach's alpha coefficient of IMFeD was 0.887 in this study. Children's sleep disturbances were measured with the Children's Sleep Habits Questionnaire (CSHQ) (40), which contains 33 items and scores of every item based on caregivers' recollection of children's sleep behaviours occurring over a recent week. Higher total scores indicate greater sleep disturbances, and Cronbach's alpha value was 0.668 in this study. Autistic tendencies of preschool children were assessed using the Chinese version of the Clancy Autism Behaviour Scale (CABS) (41). The CABS contains 14 items, and higher total scores indicate greater autistic tendencies. Cronbach's alpha coefficient of the CABS was 0.809 in this study.

#### Prenatal and Perinatal Factors

Data on prenatal and perinatal factors were obtained from the questionnaire including gestational age (weeks), birth weight (g), delivery mode, pregnancy complications (hypertension, diabetes, and anaemia), maternal prenatal and postnatal emotional symptoms, and exclusive breastfeeding. Preterm birth was defined as a gestational age < 37 weeks. Low birth weight was defined as infants with a birth weight below 2,500 g.

#### Family Factors

Information on region of kindergartens, parental education, parental marital status, parenting attitude, children's sibling, left-behind experience, and monthly household income (RMB) was collected from the questionnaire by the caregivers' response on the questionnaire. Left-behind experience was defined as the experience that children who had been left behind by one or both parent(s) who migrated to other cities for employment for more than 6 consecutive months. Contact physical abuse (hit or slapped the child on the bottom; hit or slapped the child on the hand, arm, or leg; pushed or shook the child, pulled the child's ears or other parts of the body; kicked the child very hard with the foot; hit or slapped the child on the face or head; hit or slapped the child on the bottom with an object; and hit the child very hard with fist) was assessed through the caregivers reporting the experience in the previous year. If one form of physical abuse was mentioned, the contact physical abuse variable was defined as “yes.” Caregiver's anxiety symptoms were evaluated using the Self-Rating Anxiety Scale (SAS) (42). The SAS contains 20 items in total, and the sum score of all items is the raw score. In the present study, the standard score which was calculated by multiplying 1.25 by the raw score was used. Higher standard scores of anxiety show a higher level of anxiety symptoms. Cronbach's alpha coefficient of the SAS was 0.82 (42).

### Dependent Variables

Anxiety symptoms of preschool children were assessed using the Chinese version of the Spence Preschool Anxiety Scale (SPAS) (22). This scale has displayed good reliability and validity in a sample of Chinese preschool children (22, 36). The scale is a 28-item anxiety symptoms measure for preschool children. The SPAS includes 5 subscales: separation anxiety disorder, physical injury fears, social phobia, obsessive-compulsive disorder, and generalised anxiety disorder. Each subscale item was scored on a 5-point scale ranging from 0 (not at all true) to 4 (very often true). The scores of the total scale and each subscale could be obtained by calculating the sum of all relevant items. The total scale score was ≥48 having significant anxiety symptoms (43). Children with scores falling in the upper quartile of the distribution were deemed as having significant subtypes of anxiety symptoms, and the cutoff value was separation anxiety disorder ≥6, physical injury fears ≥12, social phobia ≥7, obsessive-compulsive disorder ≥6, and generalised anxiety disorder ≥5, respectively. In our study, Cronbach's alpha value for the subscales ranged from 0.613 to 0.728 and that for the total scale was 0.903.

### Statistical Analysis

Continuous variables are described as the mean ± standard deviation (SD), and differences between groups were tested by Student's *t* tests or analysis of variance. Categorical variables were expressed as frequencies and percentages, and the chi-squared (χ^2^) test was utilised for comparisons. Logistic regression analyses were conducted to explore the associations of children's total anxiety symptoms and anxiety symptoms in five subtypes with individual, prenatal and perinatal, and family factors; and odds ratio (OR) and its 95% confidence interval (*CI*) were reported. To select the optimal regression equation, variables with *p* < 0.05 were entered into multivariate logistic regression models and those with *p* < 0.10 were retained in the final model using the forward stepwise manner. The 10-fold cross-validation method was used to assess the performance (area under the curve (AUC), sensitivity, specificity, and accuracy) of the multivariate regression model to avoid overfitting. The entire dataset was divided into 10 non-overlapping equivalent subsets; nine subsets were used to produce a prediction model, and the model was validated with the last subset. The procedure was repeated 10 times to ensure that each subset was applied once for model verification. Cross-validation was performed using the “caret” and “pROC” package in R software (Version 4.1.0, R Foundation for Statistical Computing, Vienna, Austria). Other analyses were conducted using SPSS software version 23.0 (SPSS Inc., USA). All *p*-values < 0.05 (two-tailed test) were considered to be statistically significant.

## Results

### Sample Characteristics

A total of 3,802 questionnaires were issued; after checking for completeness and logic, 3,636 effective questionnaires were finally collected. Comparisons of some variables based on preschool children's anxiety symptoms are shown in Table 1. Of the 3,636 preschool children, the mean age was 4.5 ± 0.9 years; 45.8% were girls. The prevalence of anxiety symptoms was higher in girls than in boys. Significant differences between subjects displaying significant anxiety and those not displaying significant anxiety were detected in child dietary habits, sleep disturbances, and autistic tendencies; prenatal and postnatal emotional symptoms, pregnancy complications; and region of kindergartens, parental education, parenting attitude, children's sibling, left-behind experience, contact physical abuse, and caregivers' anxiety symptoms.

**Table 1:** Individual, prenatal and perinatal, and family factors by preschool children's anxiety symptoms.

**Variables**		**Sample**	**Significant anxiety symptoms** ***n*** **(%) or means ± SD**		***p-*value**
			**Yes (n = 330)**	**No (n = 3,306)**	
**Individual characteristics**					
Age	Year	3,636	4.6 ± 0.9	4.5 ± 0.9	0.295
Gender	Boys	1,970	157 (8.0)	1,813 (92.0)	**0.012**
	Girls	1,666	173 (10.4)	1,493 (89.6)	
Body mass index	Normal	3,029	275 (9.1)	2,754 (90.9)	0.989
	Overweight or obesity	607	55 (9.1)	552 (90.9)	
Dietary behaviour	Score	3,636	62.7 ± 13.0	70.2 ± 11.5	**<0.001**
Sleep disturbances	Score	3,636	53.8 ± 6.9	49.0 ± 6.7	**<0.001**
Autistic tendencies	Score	3,636	10.9 ± 4.7	7.3 ± 4.5	**<0.001**
**Prenatal and perinatal factors**					
Delivery mode	Vaginal delivery	2,092	205 (9.8)	1,887 (89.2)	0.077
	Caesarean section	1,544	125 (8.1)	1,419 (91.9)	
Low birth weight	No	3,489	313 (9.0)	3,176 (91.0)	0.284
	Yes	147	17 (11.6)	130 (88.4)	
Preterm birth	No	3,517	317 (9.0)	3,200 (91.0)	0.475
	Yes	119	13 (10.9)	106 (89.1)	
Prenatal emotional symptoms	Pleasure or fair	3,415	289 (8.5)	3,126 (91.5)	**<0.001**
	Poor	221	41 (18.6)	180 (81.4)	
Postnatal emotional symptoms	Pleasure or fair	3,330	290 (8.7)	3,040 (91.3)	**0.011**
	Poor	306	40 (13.1)	266 (86.9)	
Pregnancy complications	No	3,150	274 (8.7)	2,876 (91.3)	**0.044**
	Yes	486	56 (11.5)	430 (88.5)	
Exclusive breastfeeding	Yes	1,979	179 (9.0)	1,800 (91.0)	0.943
	No	1,657	151 (9.1)	1,506 (90.9)	
**Family factors**					
Region of kindergartens	Hefei	1,056	76 (7.2)	980 (92.8)	**0.026**
	Fuyang	1,595	164 (10.3)	980 (89.7)	
	Ma'anshan	985	76 (9.1)	980 (90.9)	
Highest household education	Middle school and below	1,754	190 (10.8)	1,564 (89.2)	**<0.001**
	High school and above	1,882	140 (7.4)	1,742 (92.6)	
Sibling	Yes	2,492	242 (9.7)	2,250 (90.3)	**0.049**
	No	1,144	88 (7.7)	1,056 (92.3)	
Left-behind experience	No	2,173	166 (7.6)	2,007 (92.4)	**<0.001**
	Yes	1,463	164 (11.2)	1,299 (88.8)	
Parental marital status	Married	3,515	315 (9.0)	3,200 (91.0)	0.196
	Divorced/widowed	121	15 (12.4)	106 (87.6)	
Monthly household income (RMB)	<5,000	1,611	154 (9.6)	1,457 (90.4)	0.365
	≥5,000	2,025	176 (8.7)	1,849 (91.3)	
Parenting attitude	Generally accordant	1,751	120 (6.9)	1,631 (93.1)	**<0.001**
	Generally discordant	1,885	210 (11.1)	1,675 (88.9)	
Contact physical abuse	No	581	40 (6.9)	541 (93.1)	**0.045**
	Yes	3,055	290 (9.5)	2,765 (90.5)	
Caregivers' anxiety symptoms	Score	3,636	41.5 ± 10.4	35.2 ± 8.5	**<0.001**

*Bold values denote p values significant at the 0.05 level*.

### Prevalence and Scores of Anxiety Symptoms

As presented in Table 2, the mean total score of anxiety symptoms was 25.3 (SD = 15.5). A total of 330 (9.1%) children met the criteria for significant anxiety symptoms. When stratified by age, the results indicated that preschool children aged 3 years were found to score significantly higher on the separation anxiety disorder subscale (*p* = 0.017), with no significant difference in different age groups on scores of the total scale and other subscales. Significant gender differences were detected in the total anxiety scale and all subscales (*p* < 0.05).

**Table 2:** Anxiety symptoms scores (mean ± SD) for overall sample and subgroups of preschool children.

		**Stratified by age**				**Stratified by gender**		
**Anxiety symptoms**	**Total sample**	**3 year**	**4 year**	**5–6 years**	***p-*value**	**Boys**	**Girls**	***p-*value**
	**(*N* = 3,636)**	**(*N* = 1,253)**	***(N* = 1,247)**	**(*N* = 1,136)**		**(*N* = 1,970)**	**(*N* = 1,666)**	
Total score	25.3 ± 15.5	25.4 ± 15.4	24.9 ± 15.4	25.6 ± 15.6	0.539	24.2 ± 15.1	26.5 ± 15.9	**<0.001**
Separation anxiety disorder	4.4 ± 3.3	4.6 ± 3.3	4.4 ± 3.3	4.2 ± 3.3	**0.017**	4.3 ± 3.3	4.5 ± 3.3	**0.034**
Physical injury fears	8.6 ± 5.6	8.5 ± 5.5	8.6 ± 5.7	8.8 ± 5.7	0.567	8.0 ± 5.4	9.3 ± 5.8	**<0.001**
Social anxiety	4.9 ± 3.8	4.9 ± 3.8	4.8 ± 3.9	5.1 ± 3.8	0.088	4.8 ± 3.8	5.1 ± 3.9	**0.007**
Obsessive-compulsive disorder	4.1 ± 3.5	4.2 ± 3.5	4.0 ± 3.4	4.2 ± 3.5	0.543	4.0 ± 3.4	4.3 ± 3.5	**0.017**
Generalised anxiety disorder	3.2 ± 2.9	3.3 ± 2.9	3.1 ± 2.8	3.3 ± 2.8	0.094	3.1 ± 2.8	3.3 ± 3.0	**0.038**

*Bold values denote p values significant at the 0.05 level*.

### Differences of Significant Anxiety Symptoms in Individual, Prenatal and Perinatal, and Family Factors

Compared with their peers, children with significant anxiety symptoms were more likely to be girls and to have siblings; left-behind experience; poor dietary habits; more sleep disturbances; more autistic tendencies; mothers who had poor prenatal and postnatal emotional symptoms; mothers who had pregnancy complications; region of kindergartens in Fuyang; parents with middle school or less education or discordant parenting attitudes; experienced contact physical abuse; and caregivers with more anxiety symptoms (*p* < 0.05; Table 1).

### Associations Between Potential Factors and Anxiety Symptoms

The result of bivariate correlations indicated that children's gender, dietary habits, sleep disturbances, and autistic tendencies; prenatal and postnatal emotional symptoms and pregnancy complications; and parental education, monthly household income, parenting attitude, contact physical abuse, and caregivers' anxiety symptoms were associated with children's anxiety symptoms (Supplementary Table 1).

As shown in Table 3, the results of multivariate logistic regression analysis indicated that girls (OR = 1.50; *p* = 0.001), left-behind experience (OR = 1.29; *p* = 0.043), more sleep disturbances (OR = 1.05; *p* < 0.001), more autistic tendencies (OR = 1.12; *p* < 0.001), poor prenatal emotional symptoms (OR = 1.54; *p* = 0.027), and caregivers with more anxiety symptoms (OR = 1.03; *p* < 0.001) were associated with increased risks of significant anxiety symptoms. In contrast, better dietary habits (OR = 0.98; *p* < 0.001) were associated with a decreased risk of significant anxiety symptoms.

**Table 3:** Logistic regression models of significant anxiety symptoms and associated factors among preschool children.

**Variables**	**Univariate analysis**		**Multivariate analysis**	
	***OR* (95% CI)**	***p-*value**	***OR* (95% CI)**	***p-*value**
**Individual characteristics**				
Age (year)	1.07 (0.94–1.21)	0.295	-	-
Girls (vs. boys)	1.34 (1.07–1.68)	**0.012**	1.50 (1.18–1.91)	**0.001**
Overweight or obesity (vs. normal)	1.00 (0.74–1.35)	0.989	-	-
Dietary behaviour	0.96 (0.95–0.96)	**<0.001**	0.98 (0.97–0.99)	**<0.001**
Sleep disturbances	1.09 (1.08–1.11)	**<0.001**	1.05 (1.03–1.07)	**<0.001**
Autistic tendencies	1.18 (1.15–1.21)	**<0.001**	1.12 (1.09–1.15)	**<0.001**
**Prenatal and perinatal factors**				
Caesarean section (vs. vaginal delivery)	0.81 (0.64–1.02)	0.078	-	-
Low birth weight (vs. no)	1.33 (0.79–2.23)	0.285	-	-
Preterm birth (vs. no)	1.24 (0.69–2.23)	0.476	-	-
Poor prenatal emotional symptoms (vs. pleasure or fair)	2.46 (1.72–3.53)	**<0.001**	1.54 (1.05–2.27)	**0.027**
Poor postnatal emotional symptoms (vs. pleasure or fair)	1.58 (1.11–2.25)	**0.012**	-	-
Pregnancy complications (vs. no)	1.37 (1.01–1.85)	**0.044**	-	-
No exclusive breastfeeding (vs. yes)	1.01 (0.80–1.27)	0.943	-	-
**Family factors**				
Region of kindergartens in Fuyang (vs. Hefei)	1.48 (1.11–1.96)	**0.007**	-	-
Region of kindergartens in Ma'anshan (vs. Hefei)	1.30 (0.94–1.78)	0.110	-	-
High school and above (vs. middle school and below)	0.66 (0.53–0.83)	**<0.001**	0.79 (0.61–1.01)	0.058
Sibling (vs. no)	1.29 (1.00–1.67)	**0.050**	-	-
Left-behind experience (vs. no)	1.53 (1.22–1.92)	**<0.001**	1.29 (1.01–1.65)	**0.043**
Divorced/widowed (vs. married)	1.44 (0.83–2.50)	0.198	-	-
Monthly household income (RMB) ≥5,000 (vs. <5,000)	0.90 (0.72–1.13)	0.366	-	-
Discordant parenting attitude (vs. accordant)	1.70 (1.35–2.15)	**<0.001**	-	-
Contact physical abuse (vs. no)	1.42 (1.01–2.00)	**0.046**	-	-
Caregivers' anxiety symptoms	1.07 (1.06–1.08)	**<0.001**	1.03 (1.02–1.05)	**<0.001**

*Bold values denote p values significant at the 0.05 level*.

To further investigate the relevant factors of five subtypes of anxiety symptoms among preschool children, the multivariate logistic regression analyses were separately performed (Table 4). The results of the analyses showed that girls, sleep disturbances, and more autistic tendencies were significantly associated with increased risks of all anxiety subtypes. Better dietary habits were associated with a decreased risk of all anxiety subtypes except for obsessive-compulsive disorder. Poor prenatal emotional symptoms increased the risks of generalised anxiety disorder, and poor postnatal emotional symptoms enhanced the odd of separation anxiety disorder. Pregnancy complications increased the risk of obsessive-compulsive disorder. The region of kindergartens in Ma'anshan was associated with increased risks of all anxiety subtypes except for separation anxiety disorder. Left-behind experience was at increased risk of physical injury fears. In addition, caregiver anxiety symptoms were significantly associated with increased odds of all anxiety subtypes.

**Table 4:** Multivariate logistic regression models anxiety symptoms in different subtypes and associated factors among preschool children.

	**Separation anxiety**		**Physical injury fears**		**Social anxiety**		**Obsessive-compulsive**		**Generalised anxiety**	
**Variables**	**disorder**						**disorder**		**disorder**	
	***OR* (95% CI)**	***p-*value**	***OR* (95% CI)**	***p-*value**	***OR* (95% CI)**	***p-*value**	***OR* (95% CI)**	***p-*value**	***OR* (95% CI)**	***p-*value**
**Individual characteristics**										
Girls (vs. boys)	1.29 (1.09–1.51)	**0.002**	1.75 (1.49–2.06)	**<0.001**	1.36 (1.15–1.60)	**<0.001**	1.27 (1.10–1.47)	**0.001**	1.25 (1.05–1.49)	**0.014**
Dietary behaviour	0.99 (0.98–0.99)	**<0.001**	0.99 (0.98–1.00)	**0.007**	0.99 (0.98–1.00)	**0.008**	−	−	0.99 (0.98-0.99)	**<0.001**
Sleep disturbances	1.05 (1.03–1.06)	**<0.001**	1.04 (1.03–1.05)	**<0.001**	1.02 (1.01–1.04)	**<0.001**	1.01 (1.00–1.02)	**0.032**	1.03 (1.02-1.05)	**<0.001**
Autistic tendencies	1.12 (1.10–1.14)	**<0.001**	1.06 (1.04–1.08)	**<0.001**	1.16 (1.13–1.18)	**<0.001**	1.10 (1.09–1.13)	**<0.001**	1.15 (1.12-1.17)	**<0.001**
**Prenatal and perinatal factors**										
Poor prenatal emotional symptoms (vs. pleasure or fair)	−	−	−	−	−	−	−	−	1.42 (1.03-1.95)	**0.031**
Poor postnatal emotional symptoms (vs. pleasure or fair)	1.38 (1.05–1.80)	**0.019**	−	−	−	−	−	−	−	-
Pregnancy complications (vs. no)	−	−	−	−	−	−	1.33 (1.09–1.64)	**0.006**		
**Family factors**										
Region of kindergartens in Fuyang (vs. Hefei)	−	−	1.08 (0.89–1.32)	0.448	0.95 (0.78–1.16)	0.596	1.16 (0.98-1.39)	0.090	1.17 (0.94-1.45)	0.161
Region of kindergartens in Ma'anshan (vs. Hefei)	−	−	1.41 (1.14–1.76)	**0.002**	1.41 (1.14–1.76)	**0.002**	1.45 (1.19–1.76)	**<0.001**	1.45 (1.14-1.85)	**0.002**
Left-behind experience (vs. no)	−	−	1.26 (1.07–1.49)	**0.005**	−	−	−	−	−	**−**
Caregivers' anxiety symptoms	1.03 (1.02–1.04)	**<0.001**	1.02 (1.01–1.03)	**<0.001**	1.04 (1.03–1.05)	**<0.001**	1.01 (1.01–1.03)	**0.003**	1.04 (1.03-1.05)	**<0.001**

*Bold values denote p values significant at the 0.05 level*.

**Figure 1:**
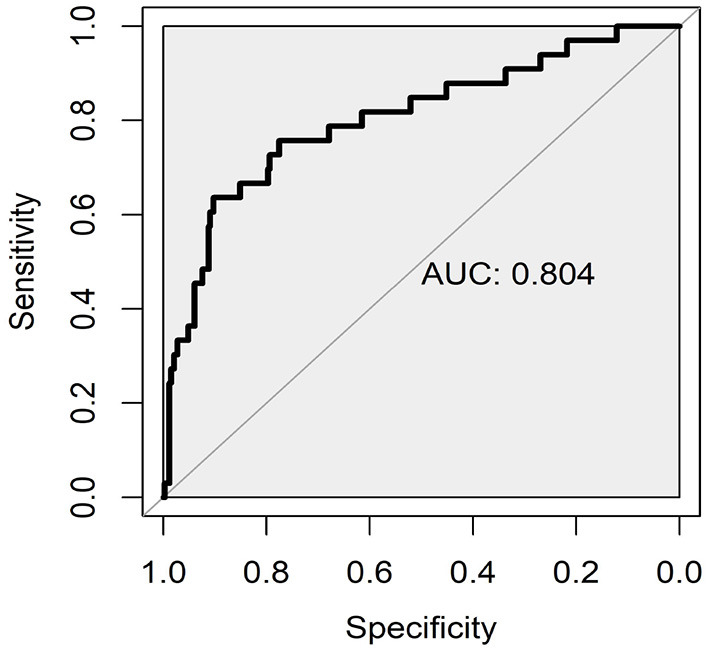
Receiver operating characteristic curves (ROC) for the predictive performance of regression model of anxiety symptoms.

The receiver operator characteristic (ROC) curve based on the highest accuracy of 10-fold cross-validation for the regression model of anxiety symptoms is presented in Figure 1. The result of 10-fold cross-validation indicated that the mean AUC, sensitivity, specificity, and accuracy were 0.78 (95% CI: 0.76–0.80), 70.45% (95% CI: 61.60–79.31%), 78.18% (95% CI: 71.50–84.87%), and 71.15% (95% CI: 63.67–78.64%), respectively. The multivariate regression models for most subtypes of anxiety symptoms demonstrated acceptable discriminative accuracy (Supplementary Table 2).

## Discussion

Due to the scarcity of large sample data regarding preschool children's anxiety symptoms in China, we conducted this cross-sectional study, including 3,636 preschool children, to explore the prevalence of anxiety symptoms and comprehensive factors. The present study found that 9.1% of child participants had significant anxiety symptoms, which was within the reported prevalence range of 7.5–22.2% from European countries and America (2–4, 24, 44), and higher than the rate of 3.3% in one Chinese study (36), but lower than that of other two Chinese studies with rates of 14.1 and 15.2% (35, 43). The wide range of prevalence rates for anxiety may be due to the populations' inherent population differences among countries and various assessment scales being used in different studies. These prevalence rates indicate that the anxiety of preschool children cannot be ignored.

Regarding individual characteristics, one interesting finding was that significant gender differences were detected for the scores of total and subtypes of anxiety symptoms. This finding was supported by one study, which has indicated that girls are affected significantly more often than boys by anxiety disorder, and gender differences might begin at preschool age (24). However, Franz found that girls reported significantly more separation anxiety disorder than boys did (2). On the contrary, no significant gender differences are detected for preschool children's anxiety in some other studies (22, 23). In contrast to gender differences, age differences only partially appeared. Although previous studies have indicated that younger children display higher scores of anxiety symptoms than older children (21, 22), the present study showed that 3-year-old children had higher scores of separation anxiety disorder, which may be attributed to their transition from the family to kindergarten. In China, most children, particularly the 3-year-old children, have just entered kindergarten. These children may experience a fear of separation as they attempt to adapt to the kindergarten life. The reasons for this separation anxiety are ambiguous, and more studies on the development and the mechanism of gender and age differences are needed for further investigation.

In addition, this study confirmed the associations between emotional and behavioural problems and anxiety among preschool children. The findings demonstrated that poor dietary habits, sleep disturbances, and autistic tendencies were associated with anxiety symptoms in preschool children. Indeed, the notion that problematic behaviours are often comorbid with mental disorders has been raised over the years (10, 45, 46). One study among 2- to 6-year-old children has indicated that anxiety symptoms are associated with a higher score of dietary behavioural problems (45). Sleep disturbances (such as late bedtime and short sleep duration) affect problematic behaviours including anxious behaviour in 5-year-old children (47). A longitudinal study has shown that infant sleep disturbances at 2 and 24 months predict anxiety symptoms at 3 years, and a bidirectional association with anxiety symptoms preceding later sleep problems seems to exist (25). Recent research has reported that anxiety symptoms are present in preschool children with autism spectrum disorder more than in typically developing children (45). In addition, a nationwide twin cohort study from Sweden discovers positive relationships between autistic-like traits and anxiety symptoms (48). This study provides more evidence on the effects of emotional and behavioural problems on preschool children's anxiety.

When referring to prenatal and perinatal factors, the results of univariate analysis revealed that poor prenatal and postnatal emotional symptoms and pregnancy complications were associated with significant anxiety symptoms in preschool children, and poor prenatal emotional symptoms were still statistically significant in multivariate analysis. Early exposure to adversities particularly during pregnancy may affect the development of the child's brain and mental health (30, 49). In line with this study, previous research has shown that children exposed to prenatal and postnatal adverse emotional symptoms are at higher risks of anxiety (27, 50). Evidence has indicated that prenatal stress has programming effects on the foetal hypothalamic–pituitary–adrenal (HPA) axis (51). Alterations in the foetal HPA axis can be long-term; therefore, the offspring may have an elevated risk of psychiatric disorders in childhood (52). Besides, associations between prenatal/perinatal risk factors and children's anxiety disorders could be mediated through parenting and family functioning or alterations in infant brain structure and function (53, 54). In addition, studies have indicated that prenatal and perinatal adversities have negative effects on mother–infant interactions, infant temperament, sleep, mental development, autism, and internalising behavior (55, 56). It should be noted that prenatal/perinatal factors and some individual factors reported above are prone to coexistence and accumulation, which can result in child anxiety.

Family played an important role in child development. A recent meta-analysis has shown that left-behind children (LBC) have an 85% higher risk of anxiety than non-LBC (57). Consistent with the literature, the results of this study revealed that LBC had a 29% increased risk of anxiety and a 46% increased risk of social anxiety. Parental labour migration and the resulting parent–child separation is common in China and leads to changes in the family environment (58). Although parental labour migration may bring economic benefits for families, it may generate hidden costs for the health of LBC (57, 59). Given that parental labour migration can sometimes not be avoided, multidimensional interventions including proactive policies and protective factors are warranted to prevent LBC from adverse health outcomes. Additionally, the results of this study suggested that caregivers' anxiety symptoms were positively associated with preschool children' anxiety symptoms. This finding is supported by theoretical models of the intergenerational transmission of anxiety, which indicates multiple pathways for the transmission of anxious cognition and behaviours (60, 61). Research has reported that caregivers or parents seem to create an anxiety-rearing environment by modelling anxious behaviours, exerting too much control over their children, and promoting anxious cognitions through the use of verbal threats, which fosters anxiety in the offspring (13, 60–63). Overall, more prospective studies are needed to clarify the association between caregivers' anxiety symptoms and preschool children's anxiety symptoms and relevant mechanistic research may benefit prevention and treatment intervention programs for anxiety.

While the underlying mechanisms of anxiety disorders are not fully understood, studies on animals and humans suggest that they are multifactorial disorders caused by the interaction between genetic and environmental factors. Researchers have proposed the genetic and epigenetic risks in the aetiology of anxiety disorders, especially in the case of familial aggregation (64, 65). Available data suggest that first-degree relatives of patients are more susceptible to developing an anxiety disorder compared with relatives of the healthy control group (20). Meanwhile, several risk genes contributing to the development of anxiety disorders have been identified (66). However, genetic factors do not act in isolation in anxiety disorders but rather interact with environmental factors. The interaction between genetic and environmental factors has been explained by epigenetic mechanisms in recent years (67, 68). The review by Babenko et al. (68, 69) concluded that prenatal stress can trigger epigenetic changes in the placenta and brain and then induce powerful influences on offspring mental health in human studies and animal models. Moreover, one study has demonstrated that effects of prenatal depressive symptoms and socioeconomic status on foetal brain development are partially modulated by genetic risk, which further confirmed gene–environment interdependence (70). Although this study included individual internal and external factors, genetic factors were limited. Future studies should pay more attention to the interaction between genetic and environmental factors.

One strength of this study is the larger sample size of preschool children and the age-appropriate validated measurement tools to assess children's anxiety symptoms. Another strength is that this study comprehensively explored factors at multiple levels of social ecology including individual, prenatal and perinatal, and family factors, which might provide a more holistic understanding of influencing factors for anxiety symptoms among preschool children. Despite these strengths, several limitations of this study should be noted. First, this study was based on the responses of a parental or other primary caregiver on a questionnaire, and prenatal and perinatal factors were retrospectively collected; hence, the possibility of reporting and recall bias cannot be eliminated. Second, preschool children were only selected from Anhui Province and a convenience sample. Thus, the external validity of the results and generalizability to the general population are partially limited. Third, this study employed a cross-sectional design, making it difficult to identify a causal relationship. Besides, in this study, these factors from pregnancy to early childhood occur sequentially or display coexistence and accumulation, and we cannot clarify their relationships. Although we applied relevant optimal models to control confounding factors, we still cannot clarify whether the interactions of the various factors amplify or attenuate the risk of child anxiety. Future longitudinal studies are required to examine causal pathways in these relationships and to determine the potential effects and interactions of prenatal, perinatal, and family factors on anxiety symptoms. Additionally, research on the underlying mechanism is important to implement prevention and intervention programs for anxiety.

In conclusion, anxiety symptoms in preschool children were prevalent, particularly in girls. Many factors from pregnancy to preschool age involving children's poor dietary habits, sleep disturbances, autistic tendencies, left-behind experience, maternal poor pregnancy emotional symptoms, and more caregiver anxiety symptoms were associated with significant anxiety symptoms among preschool children. The findings highlighted the importance of understanding early-life risk factors for anxiety, and efficient prevention and intervention strategies should be implemented in early childhood even to the perinatal period.

## Data Availability Statement

All data generated or analyzed during this study are included in this article and Supplementary Material. Further inquires can be directed to the corresponding author.

## Ethics Statement

The studies involving human participants were reviewed and approved by Ethics Committee of Anhui Medical University. Written informed consent to participate in this study was provided by the participant's legal guardian/next of kin.

## Author Contributions

XD designed this study and wrote the manuscript. NL, WS, HW, QS, XG, ML, QQ, LS, and MC recruited the participants, administered the assessment, and undertook the data collation and analysis. JW and YS guided and revised the writing. All authors listed have read the final manuscript and approved it for publication.

## Funding

This work was supported by the National Natural Science Foundation of China (Grant Number: 81872704) and the Natural Science Foundation in Higher Education of Anhui (Grant Number: KJ2020A0208).

## Conflict of Interest

The authors declare that the research was conducted in the absence of any commercial or financial relationships that could be construed as a potential conflict of interest.

## Publisher's Note

All claims expressed in this article are solely those of the authors and do not necessarily represent those of their affiliated organizations, or those of the publisher, the editors and the reviewers. Any product that may be evaluated in this article, or claim that may be made by its manufacturer, is not guaranteed or endorsed by the publisher.
