# Supplementary material for: Individual, Prenatal, Perinatal, and Family Factors for Anxiety Symptoms Among Preschool Children
Source: Front Psychiatry. 2021 Dec 20;12:778291. doi: 10.3389/fpsyt.2021.778291 (PMC8721098; doi:10.3389/fpsyt.2021.778291)
Supplement: Supplementary file 1 [file Data_Sheet_1.doc]

**Supplementary Table 1** Correlation matrix for of the study variables (N=3636).

| Variables | 1 | 2 | 3 | 4 | 5 | 6 | 7 | 8 | 9 | 10 | 11 | 12 | 13 | 14 | 15 | 16 | 17 | 18 | 19 | 20 | 21 | 22 | 23 |
| --- | --- | --- | --- | --- | --- | --- | --- | --- | --- | --- | --- | --- | --- | --- | --- | --- | --- | --- | --- | --- | --- | --- | --- |
| 1. Children’s anxiety symptoms | 1.00 | .01 | .07^**^ | .01 | .26^**^ | .32^**^ | .43^**^ | -.01 | .01 | -.02 | .08^**^ | .08^**^ | .08^**^ | -.01 | .03 | -.05^**^ | .02 | .03 | .02 | -.07^**^ | .11^**^ | .14^**^ | .32^**^ |
| 1. Age |  | 1.00 | .01 | -.06^**^ | .08^**^ | -.09^**^ | -.09^**^ | .06^**^ | -.01 | -.03 | .01 | -.02 | -.05^**^ | .02 | .01 | -.10 | .01 | .08^**^ | .01 | -.05 | .01 | -.01 | .01 |
| 1. Gender |  |  | 1.00 | -.03^*^ | .01 | .01 | -.09^**^ | -.02 | .01 | -.01 | .01 | -.02 | .01 | -.02 | .03 | -.01 | .08^**^ | -.02 | .01 | -.03^*^ | .01 | -.03 | .03^*^ |
| 1. Body mass index |  |  |  | 1.00 | .08^**^ | .01 | .03 | .04^*^ | -.04^*^ | -.01 | .01 | .02 | -.02 | .03 | -.05^**^ | .05^**^ | .02 | -.03 | -.01 | .05^**^ | .01 | .01 | .01 |
| 1. Dietary behavior |  |  |  |  | 1.00 | -.37^**^ | -.31^**^ | .03^*^ | -.01 | .01 | -.09^**^ | -.09^**^ | -.06^**^ | -.04^*^ | -.01 | -.03^**^ | -.03^*^ | -.01 | -.02 | .01 | -.11^**^ | -.11^**^ | -.23^**^ |
| 1. Sleep disturbances |  |  |  |  |  | 1.00 | .30^**^ | -.03 | -.01 | -.01 | .10^**^ | .11^**^ | .08^**^ | .01 | -.06^**^ | -.03^*^ | -.01 | -.03 | .02 | -.04^*^ | .14^**^ | .11^**^ | .31^**^ |
| 1. Autistic tendencies |  |  |  |  |  |  | 1.00 | -.02 | .01 | -.03 | .10^**^ | .11^**^ | .08^**^ | .03^*^ | -.02 | -.02 | -.02 | .05^**^ | .01 | -.06^**^ | .16^**^ | .17^**^ | .30^**^ |
| 1. Delivery mode |  |  |  |  |  |  |  | 1.00 | .01 | .03 | -.01 | .01 | .08^**^ | .04^**^ | -.05^**^ | .05^**^ | -.06^**^ | -.05^**^ | -.01 | .06^**^ | .01 | .02 | -.02^**^ |
| 1. Low birth weight |  |  |  |  |  |  |  |  | 1.00 | .37^**^ | .01 | .02 | .03 | .08^**^ | .01 | -.03^*^ | .01 | .03 | .01 | .03 | -.01 | -.02 | .03^**^ |
| 1. Preterm birth |  |  |  |  |  |  |  |  |  | 1.00 | .04^**^ | .08^**^ | .05^**^ | .07^**^ | -.01 | .05^**^ | .01 | .01 | -.01 | .06^**^ | -.02 | -.02 | .02 |
| 1. Prenatal emotional symptoms |  |  |  |  |  |  |  |  |  |  | 1.00 | .45^**^ | .10^**^ | -.01 | -.04^*^ | -.04^*^ | -.01 | .01 | .06^**^ | -.03 | .10^**^ | .05^**^ | .13^**^ |
| 1. Postnatal emotional symptom |  |  |  |  |  |  |  |  |  |  |  | 1.00 | .12^**^ | .02 | -.05^**^ | .02 | -.03 | .01 | .03 | .01 | .14^**^ | .07^**^ | .15^**^ |
| 1. Pregnancy complications |  |  |  |  |  |  |  |  |  |  |  |  | 1.00 | .04^*^ | -.06^**^ | .02 | .04^*^ | .01 | -.01 | -.01 | .10^**^ | .05^**^ | .06^**^ |
| 1. Exclusive breastfeeding |  |  |  |  |  |  |  |  |  |  |  |  |  | 1.00 | .07^**^ | .01 | -.03^*^ | .01 | .05^**^ | -.02 | -.02 | -.03 | .02 |
| 1. Region of kindergartens |  |  |  |  |  |  |  |  |  |  |  |  |  |  | 1.00 | -.22^**^ | -.06^**^ | .07^**^ | .03 | -.11^**^ | -.07^**^ | -.08^**^ | -.09^**^ |
| 1. Parental education |  |  |  |  |  |  |  |  |  |  |  |  |  |  |  | 1.00 | -.15^**^ | -.23^**^ | -.05^**^ | .23^**^ | .06^**^ | .05^**^ | -.09^**^ |
| 1. Sibling |  |  |  |  |  |  |  |  |  |  |  |  |  |  |  |  | 1.00 | .02 | -.09^**^ | -.10^**^ | -.04^*^ | .01 | .05^**^ |
| 1. Left-behind status |  |  |  |  |  |  |  |  |  |  |  |  |  |  |  |  |  | 1.00 | .05^**^ | -.10^**^ | -.06^**^ | -.05^**^ | .09^**^ |
| 1. Parental marital status |  |  |  |  |  |  |  |  |  |  |  |  |  |  |  |  |  |  | 1.00 | -.09^**^ | -.02 | -.01 | .06^**^ |
| 1. Monthly household income |  |  |  |  |  |  |  |  |  |  |  |  |  |  |  |  |  |  |  | 1.00 | .02 | .04^**^ | -.05^**^ |
| 1. Parenting attitude |  |  |  |  |  |  |  |  |  |  |  |  |  |  |  |  |  |  |  |  | 1.00 | .11^**^ | .13^**^ |
| 1. Contact physical abuse |  |  |  |  |  |  |  |  |  |  |  |  |  |  |  |  |  |  |  |  |  | 1.00 | .16^**^ |
| 1. Caregivers’ anxiety symptoms |  |  |  |  |  |  |  |  |  |  |  |  |  |  |  |  |  |  |  |  |  |  | 1.00 |

Note. Bivariate correlation analyses were performed using the Pearson’s or Spearman’s correlation test based on the variable distribution.

^*^*P*<.05, ^**^*P*<.01 (two-tailed test).

**Supplementary** **Table 2** Performance indicators for models of subtypes of anxiety symptoms (Means and 95% confident intervals).

| Model for subtypes | AUC | Sensitivity (%) | Specificity (%) | Accuracy (%) |
| --- | --- | --- | --- | --- |
| Separation anxiety disorder | 0.74 (0.72-0.76) | 65.60 (61.12-70.09) | 70.00 (65.35-74.65) | 66.69 (64.46-68.93) |
| Physical injury fears | 0.69 (0.67-0.70) | 60.29 (59.20-61.38) | 68.97 (68.29-69.64) | 62.37 (61.64-63.10) |
| Social anxiety | 0.75 (0.73-0.76) | 61.14 (55.80-66.47) | 78.96 (74.30-83.61) | 65.56 (62.46-68.65) |
| Obsessive-compulsive disorder | 0.65 (0.63-0.68) | 69.25 (61.38-77.11) | 56.05 (45.81-66.29) | 64.96 (62.48-67.44) |
| Generalized anxiety disorder | 0.76 (0.74-0.78) | 64.14 (57.61-70.68) | 79.18 (73.96-84.50) | 67.22 (62.92-71.51) |

Note. AUC= area under the curve.
